# The semantics of microglia activation: neuroinflammation, homeostasis, and stress

**DOI:** 10.1186/s12974-021-02309-6

**Published:** 2021-11-06

**Authors:** Samuel C. Woodburn, Justin L. Bollinger, Eric S. Wohleb

**Affiliations:** Department of Pharmacology & Systems Physiology, University of Cincinnati College of Medicine, Cincinnati, OH USA

**Keywords:** Microglia, Inflammation, Homeostasis, Stress, Parainflammation, Depression

## Abstract

Microglia are emerging as critical regulators of neuronal function and behavior in nearly every area of neuroscience. Initial reports focused on classical immune functions of microglia in pathological contexts, however, immunological concepts from these studies have been applied to describe neuro-immune interactions in the absence of disease, injury, or infection. Indeed, terms such as ‘microglia activation’ or ‘neuroinflammation’ are used ubiquitously to describe changes in neuro-immune function in disparate contexts; particularly in stress research, where these terms prompt undue comparisons to pathological conditions. This creates a barrier for investigators new to neuro-immunology and ultimately hinders our understanding of stress effects on microglia. As more studies seek to understand the role of microglia in neurobiology and behavior, it is increasingly important to develop standard methods to study and define microglial phenotype and function. In this review, we summarize primary research on the role of microglia in pathological and physiological contexts. Further, we propose a framework to better describe changes in microglia1 phenotype and function in chronic stress. This approach will enable more precise characterization of microglia in different contexts, which should facilitate development of microglia-directed therapeutics in psychiatric and neurological disease.

## Background

In the early 1900s, Pío del Río-Hortega described a new type of phagocytic brain cell, of mesodermal origin, that he termed “microglia” [1–3]. Decades later, microglia were recognized as macrophages, evidenced by morphological similarities to peripheral macrophages and their recognition by antisera against common monocyte markers [4–7]. Still, microglia are unique among macrophages. Unlike their fetal liver or bone marrow-derived relatives, microglia arise from embryonic yolk sack progenitors and maintain transcriptional and functional identities distinguishing them from other macrophages which may infiltrate the brain under pathological conditions [8–15]. As such, microglia are considered brain-resident macrophages and are uniquely suited to regulate neural homeostasis and behavior. In particular, recent studies indicate that microglia can modulate neuronal activity, facilitate learning, and shape social behavior [16–24].

**Fig. 1:**
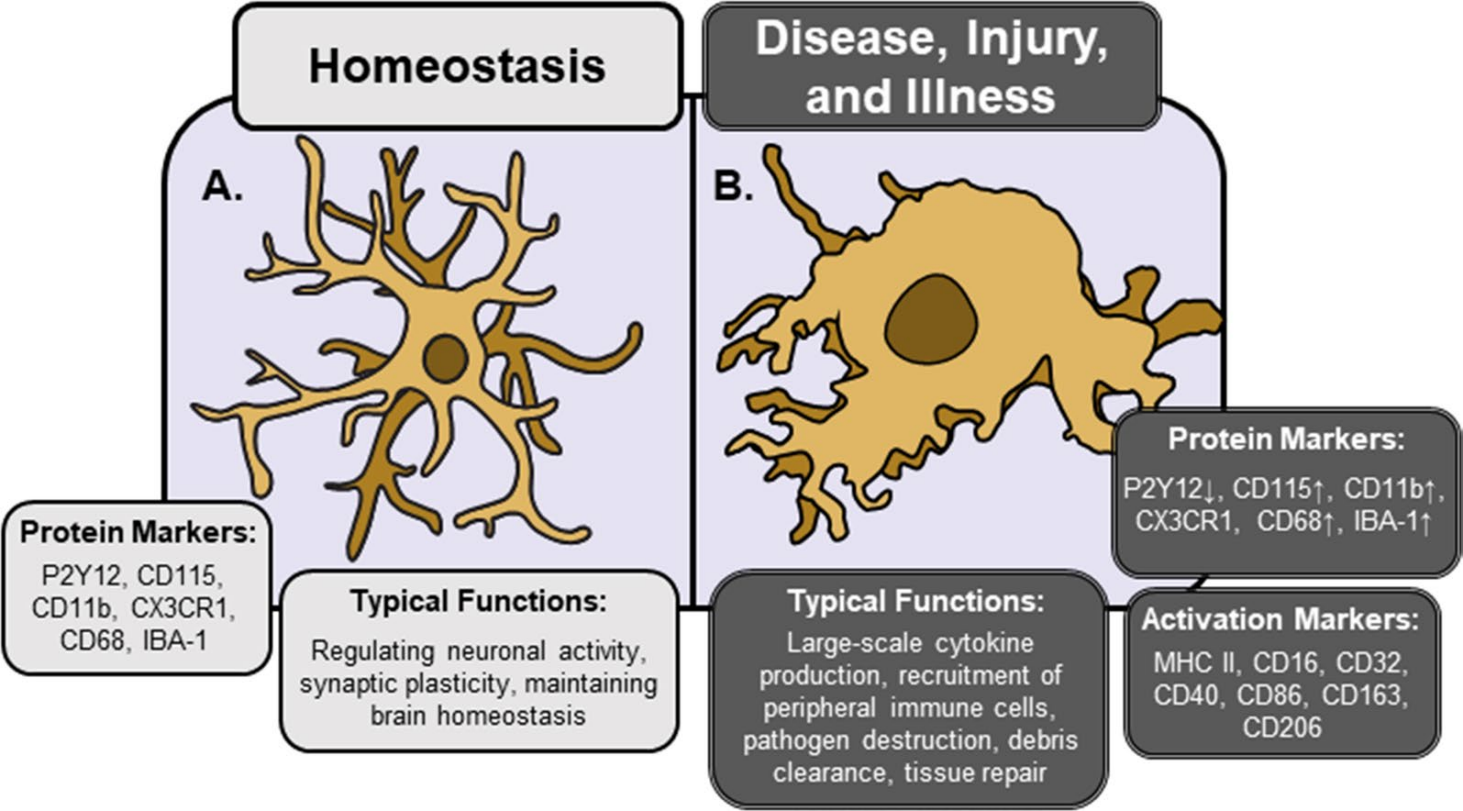
Phenotypic distinctions between homeostatic and “activated” microglia. Under typical conditions (**A**), microglia display a ramified morphology and a unique pattern of gene expression, making them identifiable through a number of different techniques. However, a variety of immunogenic stimuli can elicit dramatic morphological and functional changes in microglia. Immunologically activated microglia (**B)** possess an amoeboid morphology; marked by increased soma size and less ramified processes. Markers for microglia activation vary widely depending on the type and severity of insult, but surface proteins associated with classical immune functions (e.g., antigen presentation, phagocytosis) tend to be increased, while those associated with homeostasis may be reduced. Functions of activated microglia are also highly variable, with the same cells potentially driving tissue damage and repair processes simultaneously. Morphological features may not correspond to assumed functional roles. Nonetheless, classical immune functions of activated microglia include phagocytosing cellular debris, large increases in cytokine signaling, recruitment of peripheral immune cells to the parenchyma, and destroying infected or dying cells

The underappreciated role of microglia in physiological conditions contrasts earlier work, which mainly focused on microglial function in pathological contexts such as injury and disease, where neuronal death or degeneration is observed [25, 26]. These initial studies characterized neuro-immune responses in such contexts and clearly defined the term “neuroinflammation,” which was guided by four primary features of peripheral inflammation: macrophage (microglial) activation, increased cytokines and chemokines, recruitment of peripheral immune cells, and local tissue damage [25, 27]. These studies also demonstrated that microglia and peripheral immune cells have dual roles: driving neuroinflammation and subsequent pathology, as well as resolving neuroinflammation and repairing the nervous system [28, 29]. Adding to the complexity of neuro-immune systems, these roles do not likely represent discrete functional states in vivo. Indeed, there is ample evidence that inflammation simultaneously drives tissue damage and repair [25, 30–33]. Nonetheless, these functions collectively represent various facets of microglia activation, which is classically identified by substantial increases in cytokine production, the adoption of an amoeboid morphology, and changes in relevant protein markers (Fig. 1.) [34–37]. It should be noted that there is ongoing refinement of approaches to characterize macrophage/microglia activation [for a comprehensive overview, see: [25, 30, 32, 33]. There are limitations to these definitions because biological systems do not fit cleanly into well-defined categories. As a result, terms such as “microglia activation” and “neuroinflammation” have broadened over the years to include most immune-related processes in the nervous system. While this has occurred in multiple areas of study, this review will focus on the functional states of microglia and their contributions to the neurobiology of stress.

Accumulating evidence indicates that microglia play a role in the synaptic and behavioral changes associated with chronic stress [38, 39]. However, there remain fundamental issues in the conceptual framework used to integrate immunological concepts with stress research. Indeed, the language used to study peripheral macrophages in the context of infection is not adequate to describe microglial function in chronic stress; especially considering the vast biological differences between these two contexts. Despite this, researchers often use the term “neuroinflammation” to describe immune-related changes caused by chronic stress, which prompts comparisons to tissue injury or disease states and ultimately hinders our understanding of stress effects on microglia. This is significant because preclinical stress models are often used to gain insight into the neurobiology of psychiatric disorders. Thus, simplifying the role of microglia and neuro-immune systems in these models may lead to spurious therapeutic targets that ultimately hinder further clinical research. As advanced approaches (e.g., single cell RNA-Seq) improve our understanding of microglial phenotypes and functions, it is important to define specific features of microglia across model systems and disease states. This will increase the likelihood that congruent molecular and cellular pathways will be identified, which may facilitate the development of therapeutic approaches targeting microglia. In this review, we will discuss microglial functions in pathological and homeostatic conditions; as well as highlight the need to refine terms used to describe microglia in different contexts. Moreover, we propose an alternative framework in which neuron–microglia interactions coordinate neurobiological adaptations to stress, congruent with an immune state called ‘parainflammation’ (not neuroinflammation).

### Microglia in inflammatory contexts

Inflammation is triggered by immunogenic stimuli (i.e., pathogens or tissue injury), resulting in various biochemical cascades aimed at resolving these threats to homeostasis. Consequently, tissue damage and repair processes are enhanced during inflammation, often in tandem, until it resolves through complex regulatory mechanisms. In the brain, neuroinflammation manifests as elevated levels of pro-inflammatory cytokines, macrophage (microglia) activation, peripheral leukocyte infiltration, and damage to nervous tissue [25]. Although it is becoming increasingly clear that neuro-immune interactions play a role in most facets of brain function, it is typically under circumstances of disease, injury, and infection that all four of these hallmarks are present [27, 40]. In high enough concentrations (i.e., during neuroinflammation), immunogenic molecules can “activate” microglia, causing them to adopt an amoeboid morphology, and drastically increase their production of cytokines and reactive oxygen species (ROS). However these functional changes vary based on the type of stimuli and severity of insult [41]. Thus, in order to understand microglial function in a disease context, it is important to appreciate the intricacies of neuroinflammation. To this end, we will discuss a few examples of microglial function in neuroinflammatory contexts.

#### Multiple sclerosis and experimental autoimmune encephalomyelitis

Microglia activation is observed in various central nervous system (CNS) diseases and is important for coordinating the immune system’s resources during disease-associated neuroinflammation. For example, activated microglia are the main phagocytes observed in early stage multiple sclerosis (MS) lesions. This changes over time, with recruited bone marrow-derived macrophages taking over as the lesion advances [42, 43]. Further insights come from mouse hepatitis virus (MHV) infection, which causes demyelinating disease in models of experimental autoimmune encephalomyelitis (EAE). Early after MHV infection, microglia increase expression of cytokines which suppress viral infection and signal to adaptive immune cells [44, 45]. Ultimately, this contributes to T-cell and peripheral macrophage infiltration, increased brain cytokine levels, and tissue damage; here in the form of plaques or lesions where activated macrophages are believed to contribute to demyelination [45, 46]. Recent studies demonstrate that the initial microglial response is a critical determinant of EAE severity. Depleting microglia with the colony stimulating factor 1 receptor (CSF1R) antagonist PLX5622 prior to MHV infection delayed virus clearance and was linked to reduced infiltration and activation of CD4^+^ T cells, as well as decreased production of interferon gamma (IFN-γ); a critical suppressor of viral replication [44, 47–49]. This dampened immune response was associated with an 80% increase in mortality [44, 47]. This is consistent with other studies reporting greater demyelination at peak disease and less remyelination during recovery in mice lacking microglia [47, 49, 50]. Thus, while microglia appear to play a role in cytokine production and demyelination in this model, it is evident that they also promote adaptive immune responses and aid in processes to repair tissue in EAE [45]. It remains to be seen whether microglial functions characterized in EAE precisely correspond to those occurring in patients with MS [43]. Nonetheless, this work highlights the complexities of neuroinflammation and microglial phenotypes in disease.

#### Ischemic stroke

Microglia are often first responders to brain or nerve injury. In fact, studies show that just minutes after ischemic stroke, microglia adopt an amoeboid morphology, display enhanced isolectin B4 binding, and have increased levels of compliment receptor 3 (CR3, a.k.a. CD11b) as well as major histocompatibility complex (MHC) I and II [51]. These activated microglia produce high levels of TNFα, leading to endothelial necroptosis and BBB destruction [52]. This secondary tissue damage may also contribute to the infiltration of peripheral monocytes in the brain parenchyma, where they proliferate and gradually take over as the predominant phagocytes at the site of injury [53]. Both microglia and peripheral monocytes are involved with clearing debris after stroke, which attenuates inflammation; however they may also phagocytose injured yet viable neurons, leading to greater tissue damage [54–56]. While these findings indicate that microglia drive pathology in stroke, there is evidence to suggest microglia also play a neuroprotective role. Mice lacking TREM2, a receptor highly expressed by microglia and believed to play a role in phagocytosis, show exacerbated ischemic damage after stroke [57]. Moreover, injured cells release high quantities of purines (ATP/ADP), which microglia detect and extend their processes toward via the purinergic receptor P2Y12 [58, 59]. Recent work showed that after stroke, neurons in the ischemic penumbra recruit microglial processes through a P2Y12 dependent mechanism, leading to the formation of an immunological synapse between the two cells [17]. Notably, this was attenuated in P2Y12 knockout mice and coincided with increased neuronal calcium load, disrupted cortical network function, and greater area of ischemia-induced disconnection [17]. Altogether these findings show that microglia have dual roles in driving the pathological hallmarks of neuroinflammation and preserving and repairing damaged tissue in stroke [60].

#### Alzheimer’s disease

Numerous studies demonstrate that microglia and neuroinflammation play a role in the pathogenesis of Alzheimer’s disease (AD). Genome-wide association studies have suggested that individuals with variants of immune genes such as CD33 and TREM2 are at greater risk of developing AD, and single-cell RNA sequencing studies suggest that immunologically activated microglia are present in both animal models of AD and tissue from AD patients [61–65]. Additionally, studies in human tissue found increased trafficking of peripheral leukocytes in brain tissue from AD patients, suggesting blood–brain barrier (BBB) decline and immune-driven pathology in AD [66–68]. In line with this, 5 × and 3 × FAD mice, which overexpress human mutations associated with familial AD, show increased neutrophil extravasation into the parenchyma at sites of amyloid beta (Aβ) plaques, where they release extracellular traps and IL-17. Importantly, depleting neutrophils reduced Aβ_42_ levels and was associated with better cognitive outcomes, suggesting that neutrophils play a role in AD pathogenesis [66]. Collectively, these studies point to neuroinflammation as a contributing factor in AD, however the precise role of microglia in AD is more complicated [69]. As mentioned above, mutations affecting TREM2 function are risk factors for AD, and importantly, TREM2 is involved in Aβ clearance by microglia [70, 71]. Further, stimulating microglia proliferation via TREM2 agonism in mice engineered to express one of these human polymorphisms, led to decreased plaque load and neuronal dystrophy, suggesting that stimulating immune function from microglia may be beneficial in AD [72]. This is in line with recent work which identified a subtype of disease-associated microglia (DAM), which are associated with Aβ plaques and promote their clearance through a mechanism involving TREM2 [73]. Thus, rather than suppressing immune function altogether, it may be useful to promote selective microglia responses to help restore homeostasis [74]. In contrast, other work suggests that microglia release Aβ and tau “seeds”, which exacerbate AD progression [75, 76]. Given these complexities, additional research will be required to fully appreciate the role of microglia in AD.

#### Meningitis

In cases of CNS infection, neuroinflammation caused by microglia activation is critical for detecting and eliminating pathogens, but is not without consequence [77]. For instance, bacterial infection of the subarachnoid space causes astrocytes, endothelia, and microglia to secrete large amounts of pro-inflammatory cytokines, including tumor necrosis factor alpha (TNF-α), interleukin 1 beta (IL-1β), and interleukin 6 (IL-6), which drive inflammation in the meninges [78]. These cytokines also activate endothelial integrins and stimulate chemokine secretion, promoting peripheral leukocyte recruitment [79, 80]. Further, *Streptococcal pneumonia*, the microorganism most commonly associated with bacterial meningitis, can penetrate the blood–brain barrier (BBB), and microglia react directly to the bacterium’s cell wall in the parenchyma [79, 81]. In vitro models and rodent studies show that components of the pneumococcal cell wall cause microglia to produce nitric oxide, as well as a variety of different cytokines and chemokines, increasing leukocyte extravasation to the parenchyma [79, 82]. This leads to a predicament where on one hand, invading pathogens will trigger apoptosis via bacterial exotoxins, ultimately resulting in death [83, 84]. On the other hand, neuroinflammation caused by cytokines and ROS from the immune system can stop infection, but at the cost of associated tissue damage [78].

#### Peripheral infection

Exposure to pathogens in peripheral tissue elicits neuro-immune signaling and microglia responses that are drastically different from CNS infection. In preclinical models, viral (i.e., poly-IC) or bacterial components (i.e., lipopolysaccharide; LPS) are used to mimic peripheral infection and study associated neuro-immune interactions. This work shows that immune cells (often tissue-resident macrophages) in peripheral tissues initiate pro-inflammatory signaling cascades that affect other immune mediators. In particular, circulating cytokines promote IL-1β secretion from macrophages in circumventricular organs, as well as perivascular macrophages and endothelia of the neurovasculature. These pro-inflammatory signals are transmitted to and propagated by microglia in the parenchyma. [85–87]. These microglia responses mediate important physiological (e.g., fever) and behavioral (e.g., lethargy) components of sickness that promote survival [88]. This form of microglial activation is transient and occurs without causing overt neuropathology [41, 88–92]. Recent reports indicate that peripheral administration of LPS can cause transient BBB disruption and microbleeds in the brain [90, 92]. These neuro-immune interactions may contribute to the observed changes in inflammatory mediators in the brain, but further studies are needed to define these mechanisms. It is important to emphasize that peripheral infection alters microglia function indirectly, as viral (i.e., poly-IC) or bacterial components (i.e., lipopolysaccharide; LPS) do not typically reach the brain parenchyma, and that effects of LPS will vary depending on the route of administration, dose, and duration of exposure [93]. Thus, studies using immune challenges, such as LPS, need to consider how neuro-immune pathways (e.g., vagus nerve; endothelial cells) transmit immune signals to microglia and the brain, and whether peripheral inflammation in these models truly drives neuroinflammation as well.

### Microglia in non-inflammatory contexts

Foundational to the idea of discrete macrophage activation states is the notion that these cells show limited activity or exist in a “resting” state prior to activation. Keeping with this, many studies note morphological shifts between the complex ramifications protruding from “resting” microglia in homeostasis and that of reactive or “amoeboid” microglia seen in diseased tissue [34, 35]. However, by focusing on microglia in the context of disease, these studies overlooked the dynamic nature of microglia in homeostasis. Seminal work utilized in vivo two-photon imaging in transgenic mice expressing EGFP driven by the *Cx3cr1* promoter to investigate microglial function under baseline conditions [94, 95]. These studies demonstrated that microglia display nearly constant movement, with their processes undergoing cycles of extension and retraction as they survey the extracellular space [94]. Further, the authors noted that neuronal activity appeared to influence process motility, and that many microglial processes contained pieces of phagocytized material which were gradually transported to the cell body. Thus, instead of quiescent cells in a “resting” state, this work demonstrated that microglia are constantly surveilling and utilize processes associated with immune activation (i.e., phagocytosis) in physiological conditions.

Further studies elaborated on this work and demonstrated that microglia are actively involved in neurodevelopment. In early development, neurons establish many more synaptic contacts than persist in a mature brain. As these young neurons send and receive signals to each other, synapses lacking sufficient input are gradually pruned while more active synapses are maintained [96, 97]. Interestingly, work by Stevens et al. showed that inactive projections from retinal ganglion cells (RGC) in the dorsal lateral geniculate nucleus (dLGN) were pruned in a complement-dependent manner [98]. In the periphery, the complement system comprised freely circulating proteins that, when bound to pathogens, trigger a signaling cascade which enhances the ability of macrophages to detect and phagocytose those pathogens [99–102]. Later work by Schafer et al. demonstrated that microglia mediate engulfment of inactive RGC projections within the developing LGN in a complement- and activity-dependent manner, which allowed for typical circuit development [103].

A number of mechanisms have been shown to guide microglia-mediated synaptic pruning, with inhibition of these pathways linked to atypical behavioral phenotypes [22, 104–106]. For example, mice lacking either TREM2 or CX3CR1, both receptors highly expressed by microglia, show impaired synaptic pruning during development [107]. Consequently, mice lacking either of these receptors exhibit altered brain connectivity and deficits in social behavior in adulthood. Additional studies even show compromised LTP alongside deficits in contextual fear conditioning and spatial learning memory in CX3CR1-deficient mice. [24, 108, 109]. Expanding on these developmental studies, recent work suggests that the complement system regulates microglial phagocytosis of synapses in the adult hippocampus and contributes to forgetting [110]. Meanwhile other experiments indicate that microglial phagocytosis of the extracellular matrix around hippocampal neurons promotes memory consolidation via a mechanism dependent on neuron-derived IL-33. Collectively these findings indicate that microglial phagocytosis is not only triggered by pathogens or injury, but it is critical for proper neurodevelopment and neuroplasticity as well.

A similar theme emerges in work examining purine (e.g., ATP/ADP)-mediated chemotaxis by microglia. Accumulating evidence indicates that this process, traditionally associated with tissue damage (see previous section), guides homeostatic neuron-microglia interactions [111]. Different labs have consistently shown that stimulation of NMDA receptors on highly active neurons causes them to release ATP/ADP into the extracellular space, which is detected by microglial P2Y12 receptors [19, 105, 112–114]. Upon binding extracellular purines, G_i_ coupled P2Y12 receptors promote extension of microglial processes toward activated neurons [17, 18, 115, 116]. Studies examining neuron-microglia interactions in the visual cortex showed that visual experience caused microglia to interact with dendritic spines of V1 neurons, and that depriving animals of light during visual critical periods increased their phagocytosis of neuronal material [117]. This was driven in part by P2Y12 signaling, as disrupting P2Y12 during monocular deprivation limited microglial remodeling of V1 synapses, which in turn abrogated typical ocular dominance [105]. Interestingly, recent studies show that this process is opposed by noradrenergic signaling, as β_2_-adrenergic receptors on microglia were shown to limit experience-dependent plasticity in awake mice by inhibiting microglial surveillance [118].

Beyond guiding neuroplasticity, convergence of microglia processes toward active neurons is implicated in regulating neuronal activity through physical contact. For example, microglia have been shown to rescue the somatic potential of hyperactive neurons by physically contacting their axons, while pharmacologically inhibiting microglial detection of neuronal activity (and thus limiting their contacts with neurons) is associated with increased neuronal death [18]. Similarly, neuronal hyperexcitability induced by kainic acid provokes more severe and more frequently lethal seizures in mice lacking P2Y12 [19], further implicating microglia in regulating neuronal excitability. Perhaps the most direct evidence of this however comes from a recent study showing that upon detection, microglia catabolize extracellular ATP into adenosine, which in turn suppresses hyperactive neurons by binding to the adenosine receptor A1R [119]. This inhibitory mechanism was crucial for attenuating seizure severity in mice treated with pro-convulsive doses of a D1 agonist, demonstrating a protective role for microglia. Taken together these studies suggest ATP-mediated chemotaxis by microglia is not only important for responding to injury, but also for maintaining brain homeostasis by limiting pathological neural activity.

Another function of activated macrophages is secretion of growth factors and cytokines. This too has been observed in microglia absent neuroinflammation. For example, Parkhurst et al. showed that microglial brain-derived neurotrophic factor (BDNF) secretion was important for synaptic integrity. In these studies, microglia-specific depletion of BDNF reduced cortical expression of certain glutamate receptors, which subsequently altered NMDA and AMPA mEPSCs [20]. These deficits in synaptic function ultimately translated to reduced learning-associated spine growth and subsequent behavioral impairments in rotarod training, indicating that trophic support from microglia is an important neurobiological component of learning. Cytokines are also known to modulate synaptic plasticity under physiological conditions. Initial studies performed both in vivo and in acute hippocampal slices showed that *Il1β* transcript was increased after LTP induction, and that application of IL-1 receptor antagonist (IL-1ra) interfered with LTP maintenance [120]. Similarly, mice engineered to overexpress IL-1ra in the brain show learning deficits, reduced GluA1/2 expression, and impaired BDNF signaling [121]. It should be noted that cytokine signaling is complex, and the same molecule might have entirely different effects depending on concentration, or the presence of other cytokines [122]. Consistent with this, IL-1β has a concentration-dependent relationship with LTP. Under physiological conditions, IL-1β concentrations are relatively low and can enhance LTP and improve recall of a conditioned stimulus. In contrast, when IL-1β concentrations are high (i.e., inflammation or aging), IL-1β impairs LTP by acting at the synapse [123–125]. Other immune factors such as TNFα, IL-10, CXCL16, and CX3CL1 have also been implicated in LTP, with microglia acting either as potential sources of, or the cells affected by these molecules [126–131]. While much work is needed to fully appreciate microglial contributions to synaptic plasticity, these studies demonstrate the importance of neuro-immune interactions in homeostatic brain function.

### Microglia in psychological stress: debating neuroinflammation

Clinical and preclinical studies conducted over the past two decades demonstrate that psychological stress influences various neuro-immune pathways [38, 132]. Early work revealed that both acute and chronic stressors increase levels of certain pro-inflammatory cytokines such as IL-1β in the brain [133, 134]. Further studies specifically implicated microglia as the primary source of these cytokines, and showed that chronic stress altered microglial morphology [133, 135–138]. Collectively these changes were considered evidence of microglia activation, and therefore neuroinflammation, caused by psychological stress. However, as outlined above, neuroinflammation is characterized by macrophage activation, pro-inflammatory cytokine secretion, leukocyte recruitment, and tissue damage [25]. While there is good evidence that all four of these are present in conditions like MS, AD, stroke, and bacterial meningitis, it is not clear if the term neuroinflammation applies to stress-associated neuro-immune functions.

Recall that the effects of pro-inflammatory cytokines depend on other factors such as concentration [122]. During neuroinflammation, IL-1β and TNFα concentrations can reach hundreds or even thousands of times higher than baseline levels, and promote cell death either by directly triggering apoptotic pathways in target cells, or by enhancing other neuroinflammatory processes associated with cellular damage [139–144]. While there is evidence that stress exposure influences IL-1β and TNFα levels, the magnitude of this effect is considerably lower by comparison (< 10 times baseline) and occurs in the absence of overt tissue pathology [41, 133, 135, 145, 146]. It should also be noted that outside the contexts of injury and disease IL-1β and TNFα secretion in the brain facilitates synaptic plasticity, which is critical in maintaining homeostasis [124, 131, 147, 148]. As such, modest changes in pro-inflammatory cytokine expression may not indicate neuroinflammation and could represent an altogether different biological process.

A common finding reported in stress literature is the presence of “activated” microglia. As mentioned in the beginning of this review, the precise definition of macrophage activation is an area of ongoing debate and it is not clear what precisely constitutes an “activated”, “resting”, or “alternatively activated” macrophage or microglia [25, 30, 32, 33]. In Fig. 1, we provide some common features of microglia across immunogenic contexts, yet even these come with a multitude of caveats, as features of “activated” microglia in one context may not appear in another [34, 35, 37]. Further, as mentioned in the previous section, the idea of “activated” microglia presumes they are quiescent under typical conditions, which is not supported by recent data [149].

One of the common metrics to assess microglia activation are broad morphological shifts from small soma with ramified processes in homeostasis, to amoeboid with thicker and less complicated processes during neuroinflammation [36, 37]. Indeed initial reports indicated that stress caused microglia activation largely based on changes in morphological features [136]. However, evidence suggests that morphological changes in microglia do not define their functional state. Indeed microglia display dynamic and varied morphologies driven by local cues and these features may not overlap with assumed functions. For example, microglia in the developing subventricular zone and cortex display amoeboid morphology, yet the cytokines and growth factors they produce are essential for neuronal growth and differentiation [150, 151]. Even within the context of chronic stress, changes in microglial morphology vary between brain region, sex, species, stress paradigm, and stress duration, without specific biological or behavioral implications [136, 152–156]. In this context, it is not accurate to suggest that morphological changes in microglia alone can provide insight into their functional state. Moreover, it should be noted that these morphological analyses have been primarily reported in tissue immunolabeled with IBA-1, which does not fully reproduce microglial complexity. Indeed, studies using mice with a microglia-specific GFP reporter failed to observe such morphological changes after chronic social defeat stress, despite having more statistical power than previous reports [157]. These findings suggest that chronic stress exposure does not cause microglia activation in a way that resembles the immunological origins of the term [32, 33, 158].

Other studies have also reported that chronic stress promotes peripheral leukocyte trafficking in the brain, but this appears to be dependent on experimental approaches and context. In particular, studies using GFP^+^ bone marrow (BM)-chimeric mice showed that repeated social defeat (RSD) promotes signaling from microglia and caused infiltration of peripheral monocytes into selective brain regions. These neuro-immune interactions were associated with reduced exploratory behavior in socially defeated mice [159–161]. However, further studies using LysM-GFP^+^, CCR2^RFP^, and UBC^GFP^ reporter mice indicate that RSD promotes accumulation of peripheral myeloid cells (i.e., monocytes and granulocytes) in the perivascular space and choroid plexus; not in the brain parenchyma [157, 159, 162, 163]. These results suggest that development of the BM-chimera model enabled extravasation of myeloid cells into the brain following RSD [157, 159, 162–164]. Despite limited evidence for leukocyte trafficking in the brain, these studies did find that RSD caused a decline in BBB integrity [157, 165]. These stress-induced changes in the BBB were associated with vascular remodeling and the passage of signaling peptides, indicating that more subtle processes are involved during stress as compared to neuroinflammation [163, 165]. It is important to point out that accumulation of peripheral myeloid cells and loss of BBB integrity has been primarily observed in the RSD model. These findings are still significant, but it reinforces the fact that the context is critical; RSD is a unique paradigm as it involves aggressive encounters that can wound subordinate mice, potentially influencing their inflammatory profile [166–168]. Indeed, other stress models that use exposure to unstable or aversive environmental conditions (and lack physical confrontations or wounding) do not cause significant accumulation of peripheral macrophages in the brain [169].

In summary, while it is evident that stress engages neuro-immune pathways and alters microglia function, these effects are subtle and are not associated with immune-driven pathology. Despite these modest effects, neuro-immune interactions still play an important role in the behavioral and biological effects of chronic stress exposure. As such, a different framework is needed to accurately describe neuro-immune responses to stress.

### Parainflammation: neuro-immune adaptations to homeostatic threats

Historically, neuro-immune interactions have been framed around existing immunological concepts derived from models of pathological conditions in which all four hallmarks of inflammation are present. By contrast, the behavioral and biological sequelae of chronic stress are best described as a consequence of prolonged adaptation to homeostatic threats, which, while associated with adverse effects, is not inherently pathological [170, 171]. For example, repeated stress exposure gradually attenuates hypothalamo–pituitary–adrenocortical (HPA) axis activation in response to a familiar challenge, ultimately reducing the physiological burden of chronic glucocorticoid secretion [170]. However, adaptation to chronic stress also sensitizes HPA activation to new challenges, which, while appropriate in dangerous situations, can be disadvantageous in response to innocuous stimuli [170, 172]. Behavioral adaptations to chronic stress can be understood in a similar way. Recent work shows that social avoidance, the primary outcome of RSD in “susceptible” mice, is a learned behavior that does not generalize outside the aggressor’s strain and can be extinguished with repeated exposure in a safe environment [173]. In other words, “susceptible” mice learn to avoid an aggressor strain in a context that reliably predicts danger. Just as well, increased immobility in the forced swim test, a behavioral metric used in many other stress paradigms, might be an adaptive pro-survival behavior, as floating in an inescapable body of water conserves energy [174]. Nonetheless, these paradigms also alter behaviors such as sucrose preference, working memory, and effort-related choice [152, 175–179]. Thus, rather than overt pathophysiology (i.e., relating to disease or injury), chronic stress effects on brain and behavior can be framed as adaptations to adverse conditions, which can compromise typical function.

**Fig. 2:**
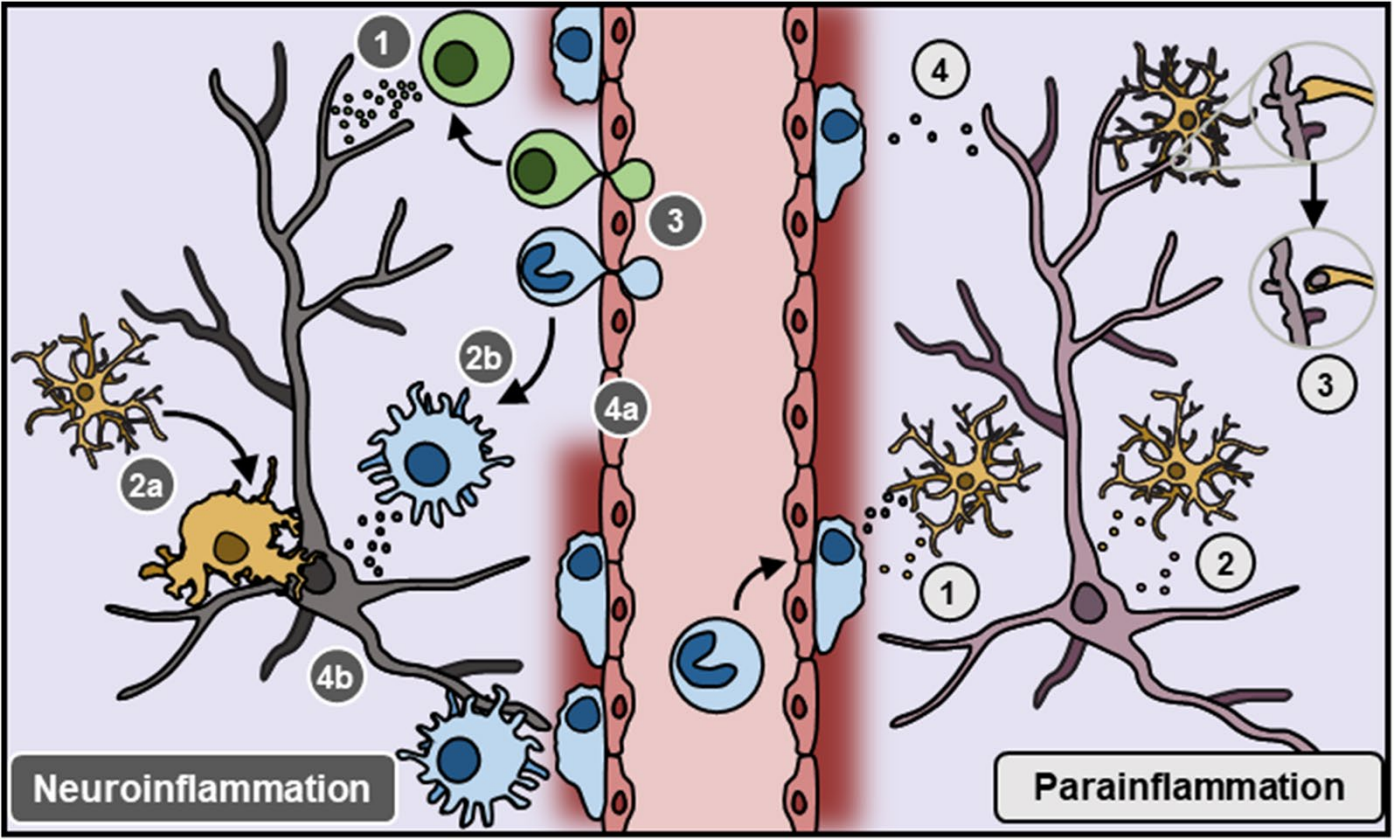
Contrast between neuroinflammation and parainflammation. Neuroinflammation (left) describes immune-driven pathology which occurs in the course of disease, injury, or infection in the brain. This tissue state can be identified by four common molecular and cellular hallmarks illustrated on the left. These are: **(1)** high levels of pro-inflammatory cytokines, **(2a)** microglia and **(2b)** peripheral macrophage activation, **(3)** infiltration of peripheral leukocytes (e.g., bone-derived monocytes, T cells) to the parenchyma, and tissue damage such as **(4a)** BBB breakdown and **(4b)** neuron death. Neuro-immune systems can also be engaged by homeostatic challenges (i.e., psychological stress), leading to an intermediate tissue state termed parainflammation (right). While a formal definition of parainflammation is yet to be widely accepted in neuroscience, neuro-immune interactions previously reported in parainflammatory contexts are shown on the right. These include: **(1)** microglial recruitment of bone-derived monocytes to the perivascular space, **(2)** changes in cytokine signaling between neurons and microglia, **(3)** microglia-mediated neuronal remodeling, and **(4)** diffusion of small signaling peptides across the BBB

With this in mind, stress-induced functional changes in microglia resemble an intermediate tissue state termed parainflammation, in which stressed or malfunctioning cells engage tissue-resident macrophages to restore homeostasis [132, 180, 181]. In this conceptual framework (Fig. 2.), chronic psychological stress disrupts homeostatic brain function, provoking changes in microglia function. In coordination with other brain cells, microglia then facilitate neurobiological adaptations by initiating immune-related processes (i.e., cytokine release, phagocytosis). Of note, these microglia-mediated processes occur independent of other peripheral immune cells and at levels relative to physiological contexts. Ultimately, this may restore brain homeostasis, but may cause important behavioral and biological consequences.

In support of this, there is growing evidence that stress-induced changes in microglial function reflect coordinated interactions between microglia and neurons (and other cells in the brain) rather than dysfunctional immune processes. For example, chronic unpredictable stress (CUS) and glucocorticoids promote CSF1 signaling in the PFC, which elicits microglial phagocytosis of neuronal elements in the medial PFC [154, 169]. Ultimately, this neuronal remodeling by microglia contributes to synapse loss in the medial PFC and associated stress-induced behavioral changes [169, 182]. Interestingly, our recent work suggests that while synapse loss persists over the course of chronic stress, microglia-mediated neuronal remodeling diminishes over time, indicating that this is a coordinated process and that spine loss is maintained by other mechanisms [152].

Further support for the concept of parainflammation in stress research comes from emerging evidence that stress-induced changes in microglia correspond to stress-induced changes in neuronal activity. Previous work showed that repeated restraint stress increased microglial proliferation in the hippocampus; however, this was blocked by treatment with the glucocorticoid receptor antagonist RU486 or the NMDA receptor antagonist MK801 prior to restraint [183]. Other studies demonstrated that acute stress exposure increased c-Fos labeling in neurons within the periaqueductal gray (PAG), and that microglia adjacent to c-Fos positive neurons displayed morphological changes [184]. Furthermore, this was distinct from morphological changes caused by LPS treatment, as LPS altered microglial morphology in the PAG regardless of c-Fos labeling on nearby neurons, indicating that microglia respond to different stimuli in these contexts. Experiments conducted in our own lab also hint that neuronal activity drives microglial function in stress. We found that treatment with diazepam limited FosB staining in the medial PFC, which corresponded to attenuated stress effects on microglia-mediated neuronal remodeling, dendritic spine density, CSF1 signaling, and behavior [155]. This is in line with research in other model systems suggesting that CSF1 is released by hyperexcitable neurons, although this remains to be directly tested in the context of chronic stress [185, 186].

Because parainflammation is an adaptive process, it is important to consider these changes within the greater context of the stress model. Seminal work in stress neurobiology demonstrated that while acute stress drives glutamate signaling in the PFC and hippocampus of male animals, this reverses as stress exposure becomes chronic [179, 187–189]. It has previously been hypothesized that, while this change is not without consequences, it ultimately reflects a metabolic shift to meet the homeostatic challenges of stress [170, 171, 190–194].

In essence, glucocorticoids released during initial stress exposure cause long-lasting potentiation of AMPA and NMDA receptor currents in pyramidal neurons, leading to increased surface expression of these receptors and improvements in working memory [187–189, 195]. However, excess or prolonged glucocorticoid and glutamate stimulation can exacerbate excitotoxic insults by increasing intracellular Ca^2+^, and high glucocorticoid levels have profound effects on metabolism by depleting available energy and inhibiting cellular growth [170, 192, 193]. Thus, over time it may become beneficial to limit the signals associated with acute stress in these regions. Keeping with this, chronic stress is associated with decreased AMPA and NMDA receptor expression, blunted EPSCs in excitatory pyramidal neurons, and impairments in working memory [179, 196]. Previous reports are also largely consistent in documenting morphological changes such as dendritic retraction and loss of apical spines in pyramidal neurons after chronic stress exposure, which would make them less susceptible to the effects of excessive Ca^2+^ [197–201].

Given this context, the changes in microglial function arising from chronic stress are quintessentially parainflammatory processes. In this framework, acute stress exposure increases excitatory neurotransmission in a subset of brain regions [187, 188]. Microglia detect this shift in neuronal activity, and may modulate it through increased (but much lower than pathological levels) cytokine secretion [124, 135, 145, 202]. As stress exposure continues, activity-dependent signals from neurons, such as CSF1, increase and direct neighboring microglia to modulate neuronal activity through structural remodeling of neurons or release of neuromodulators [169]. These parainflammatory mechanisms are also accompanied by cell autonomous mechanisms in neurons, such as dendritic retraction and degradation of glutamate receptors, which maintain this neuronal phenotype [179, 197, 200, 203]. Collectively, these coordinated neuron–microglia interactions mitigate the effects of uncontrolled glutamate signaling and promote behavioral adaptations to adverse conditions. As an inadvertent result however, these changes may also promote longer-term behavioral and physiological consequences. Thus, while parainflammatory mechanisms are not associated with overt tissue pathology (as is the case in neuroinflammation), some aspects can be considered maladaptive.

### Clinical implications

This is a semantic argument, but it is significant, as stress models are often used to inform our understanding of psychiatric conditions such as major depressive disorder (MDD). Indeed, preclinical work attributing stress effects to neuroinflammation and microglia activation are frequently cited by clinical studies claiming a similar etiology for MDD. These clinical reports indicate that some individuals with MDD have higher blood cytokine levels than controls, while others report increased PET detection of translocator protein (TSPO) during depressive episodes, which is interpreted as an indication of microglia activation and neuroinflammation [204, 205]. While this work provides evidence of altered immune signaling in MDD, much like the basic research discussed in this review, these measures do not necessarily indicate that neuroinflammation or immune dysregulation underlies the etiology of MDD.

Indeed, mild infections or experimentally induced inflammation with LPS increase blood concentrations of inflammatory cytokines to much higher levels, suggesting that peripheral immune signaling in MDD differs from that observed during inflammation [206–208]. Similarly, although TSPO has been used as a proxy for microglia activation, the functional consequences of increased TSPO are not entirely understood [209]. In fact, PET imaging studies conducted in patients with MS show no difference in TSPO expression between activated macrophages in white matter lesions and those in normal appearing white matter or in healthy controls [210]. Other studies have indicated additional confounds to consider in TSPO studies, namely that TSPO tracers are bound by vascular endothelia and TSPO levels show maximal intensity after the resolution of inflammation [211, 212]. Further, preclinical studies indicate that TSPO expression is also increased in neurons, not microglia, after periods of heightened neurotransmission [213]. These findings suggest that the utility of TSPO in assessing microglia activation is limited by its lack of cellular and temporal specificity. The role of neuroinflammation in MDD was further challenged by recent clinical studies that showed microglia from individuals with MDD have increased expression of transcripts and proteins associated with homeostatic functions, rather than markers of inflammation [214].

Our focus on this literature is not intended to dismiss a potential relationship between MDD and inflammation outright. There is strong evidence to suggest that genuine inflammatory responses can disrupt mood in patients with chronic illness or comorbid inflammatory diseases [215–217]. In addition, recent findings demonstrate that there are differences in circulating immune factors and cells in subsets of MDD patients [218]. Considering the evidence however, it seems unlikely that MDD or the psychological effects of stress can be considered truly neuroinflammatory conditions on their own. And although neuro-immune interactions have clearly been implicated in MDD, they appear to reflect an intermediate immune state rather than classic inflammation. Our understanding of immune function in MDD is still developing and further research is needed to fully appreciate its complexity. As such, hypotheses that reduce multidimensional diagnoses like MDD to pathological immune function are not likely to advance treatments for patients. This necessitates the use of conceptual frameworks that integrate parainflammatory neuro-immune interactions within a larger biopsychosocial context, as has been done in other areas of neuroscience and psychiatry [219–221].

## Conclusion

As research on the involvement of neuro-immune systems in chronic stress expands, it is imperative that our models and conceptual frameworks be adapted to relevant data. Initial studies used existing disease-based terms to define stress-associated changes in microglial function. However, preclinical research demonstrates that neuro-immune responses to stress are distinct from CNS disease, injury, or infection and should not be characterized as neuroinflammation [25, 41]. The neurobiological changes that occur with chronic stress reflect adaptations required to meet the homeostatic demands of an unstable environment. We suggest that, before concluding a given neuro-immune interaction is evidence of neuroinflammation, investigators first consider whether the hallmarks of inflammation are also present. These include: substantially increased cytokine concentrations, macrophage activation, parenchymal infiltration of peripheral immune cells, and pathological tissue damage [25]. We also urge stress researchers to consider how observed neuro-immune changes compare to other model systems within and beyond that context. It is also worth considering if these changes are entirely detrimental, or if they might reflect an adaptive, or even beneficial (i.e., eustress) response to unstable conditions [222, 223].

Distinction between pathological (inflammation) and adaptive (parainflammation) functions of neuro-immune interactions are necessary if we hope to one day leverage them for therapeutic purposes. To this end, technologies such as single-cell RNA-Seq and time-of-flight mass cytometry can lend important insights, as these approaches enables researchers to understand the heterogeneity of cellular populations and can identify molecular patterns to distinguish various functional states [73, 224, 225]. This is particularly relevant to tissue-resident macrophages, such as microglia, as they display a remarkable degree of functional plasticity [31]. Given these technical advances and appreciation for the spectrum of microglia function, we propose that it is time to shift the conceptual framework used to describe neuro-immune function in non-disease contexts, such as stress. Indeed, there is substantial evidence that neuro-immune interactions in chronic stress resemble parainflammation; an intermediate immune state where immune responses are directed toward reestablishing homeostasis [181]. In the end, this framework will enable researchers to more accurately describe the functional role of microglia in various contexts; and we are hopeful this leads to new therapeutic strategies involving microglia.

## Data Availability

Not applicable.

## Abbreviations

A1R: Adenosine A1 receptor
ADP: Adenosine diphosphate
AMPA: α-Amino-3-hydroxy-5-methyl-4-isoxazolepropionic acid
Aβ: Amyloid beta
ATP: Adenosine triphosphate
BBB: Blood–brain barrier
BDNF: Brain-derived neurotrophic factor
BM: Bone marrow
CCR2: C–C Motif Chemokine Receptor 2
CNS: Central nervous system
CR3: Compliment receptor 3
CSF1: Colony stimulating factor 1
CSF1R: Colony stimulating factor 1 receptor
CUS: Chronic unpredictable stress
CX3CL1: C-X3-C motif chemokine ligand 1
CX3CR1: C-X3-C motif chemokine receptor 1
CXCL16: C-X-C motif chemokine ligand 6
D1: Dopamine 1 receptor
dLGN: Dorsal lateral geniculate nucleus
EAE: Experimental autoimmune encephalomyelitis
EGFP: Enhanced green fluorescent protein
HPA: Hypothalamo–pituitary–adrenocortical
IFN-γ: Interferon gamma
IL-1β: Interleukin 1 beta
IL-10: Interleukin 10
IL-1ra: IL-1 receptor antagonist
IL-6: Interleukin 6
IL-33: Interleukin 33
LPS: Lipopolysaccharide
LTP: Long-term potentiation
MDD: Major depressive disorder
mEPSC: Miniature excitatory postsynaptic current
MHC: Major histocompatibility complex
MHV: Mouse hepatitis virus
MS: Multiple sclerosis
NMDA: N-Methyl-D-aspartate
PAG: Periaqueductal gray
PET: Positron emission tomography
PFC: Prefrontal cortex Poly-IC: Polyinosinic:polycytidylic acid
P2Y12: Purinergic receptor P2Y, G-protein coupled, 12
RGC: Retinal ganglion cells
ROS: Reactive oxygen species
RSD: Repeated social defeat
TREM2: Triggering receptor expressed on myeloid cells 2
TSPO: Translocator protein
TNF-α: Tumor necrosis factor alpha
UBC: Ubiquitin C

## References

[1] Boullerne AI, Feinstein DL (2020). History of Neuroscience I. Pío del Río-Hortega (1882–1945): the discoverer of microglia and oligodendroglia. ASN Neuro.

[2] del Río-Hortega BJ (2020). Pío del Río-Hortega: the revolution of Glia. Anat Rec.

[3] Prinz M, Jung S, Priller J (2019). Microglia biology: one century of evolving concepts. Cell.

[4] Murabe Y, Sano Y (1983). Morphological studies on neuroglia—VII. Distribution of “brain macrophages” in brains of neonatal and adult rats, as determined by means of immunohistochemistry. Cell Tissue Res.

[5] Murabe Y, Sano Y (1982). Morphological studies on neuroglia—VI. Postnatal development of microglial cells. Cell Tissue Res.

[6] Perry VH, Hume DA, Gordon S (1985). Immunohistochemical localization of macrophages and microglia in the adult and developing mouse brain. Neuroscience.

[7] Hume DA, Perry VH, Gordon S (1983). Immunohistochemical localization of a macrophage-specific antigen in developing mouse retina: Phagocytosis of dying neurons and differentiation in microglial cells to form a regular array in the plexiform layers. J Cell Biol.

[8] Cronk JC, Filiano AJ, Louveau A, Marin I, Marsh R, Ji E (2018). Peripherally derived macrophages can engraft the brain independent of irradiation and maintain an identity distinct from microglia. J Exp Med.

[9] Gosselin D, Skola D, Coufal NG, Holtman IR, Schlachetzki JCM, Sajti E (2017). An environment-dependent transcriptional network specifies human microglia identity. Science.

[10] Alliot F, Godin I, Pessac B (1999). Microglia derive from progenitors, originating from the yolk sac, and which proliferate in the brain. Dev Brain Res.

[11] Alliot F, Lecain E, Grima B, Pessac B (1991). Microglial progenitors with a high proliferative potential in the embryonic and adult mouse brain. Proc Natl Acad Sci U S A.

[12] Ginhoux F, Greter M, Leboeuf M, Nandi S, See P, Gokhan S (2010). Fate mapping analysis reveals that adult microglia derive from primitive macrophages. Science.

[13] Schulz C, Perdiguero EG, Chorro L, Szabo-Rogers H, Cagnard N, Kierdorf K (2012). A lineage of myeloid cells independent of myb and hematopoietic stem cells. Science.

[14] Geissmann F, Auffray C, Palframan R, Wirrig C, Ciocca A, Campisi L (2008). Blood monocytes: distinct subsets, how they relate to dendritic cells, and their possible roles in the regulation of T-cell responses. Immunol Cell Biol.

[15] Kierdorf K, Erny D, Goldmann T, Sander V, Schulz C, Perdiguero EG (2013). Microglia emerge from erythromyeloid precursors via Pu.1-and Irf8-dependent pathways. Nat Neurosci.

[16] Eyo UB, Peng J, Murugan M, Mo M, Lalani A, Xie P (2016). Regulation of physical microglia—neuron interactions by fractalkine signaling after status epilepticus. eNeuro..

[17] Cserep C, Posfai B, Lenart N, Fekete R, Laszlo ZI, Lele Z (2019). Microglia monitor and protect neuronal function via specialized somatic purinergic junctions. Science.

[18] Kato G, Inada H, Wake H, Akiyoshi R, Miyamoto A, Eto K (2016). Microglial contact prevents excess depolarization. eNeuro.

[19] Eyo UB, Peng J, Swiatkowski P, Mukherjee A, Bispo A, Wu LJ (2014). Neuronal hyperactivity recruits microglial processes via neuronal NMDA receptors and microglial P2Y12 receptors after status epilepticus. J Neurosci.

[20] Parkhurst CN, Yang G, Ninan I, Savas JN, Yates Iii JR, Lafaille JJ (2013). Microglia promote learning-dependent synapse formation through brain-derived neurotrophic factor (+ SI). Cell.

[21] Kana V, Desland FA, Casanova-Acebes M, Ayata P, Badimon A, Nabel E (2019). CSF-1 controls cerebellar microglia and is required for motor function and social interaction. J Exp Med.

[22] Kim HJ, Cho MH, Shim WH, Kim JK, Jeon EY, Kim DH (2017). Deficient autophagy in microglia impairs synaptic pruning and causes social behavioral defects. Mol Psychiatry.

[23] VanRyzin JW, Marquardt AE, Argue KJ, Vecchiarelli HA, Ashton SE, Arambula SE (2019). Microglial phagocytosis of newborn cells is induced by endocannabinoids and sculpts sex differences in juvenile rat social play. Neuron.

[24] Zhan Y, Paolicelli RC, Sforazzini F, Weinhard L, Bolasco G, Pagani F (2014). Deficient neuron-microglia signaling results in impaired functional brain connectivity and social behavior. Nat Neurosci.

[25] Estes ML, McAllister AK (2014). Alterations in immune cells and mediators in the brain: It’s Not always neuroinflammation!. Brain Pathol.

[26] Tremblay MÈ, Stevens B, Sierra A, Wake H, Bessis A, Nimmerjahn A (2011). The role of microglia in the healthy brain. J Neurosci.

[27] O’Callaghan JP, Sriram K, Miller DB (2008). Defining “neuroinflammation”: Lessons from MPTP- and methamphetamine-induced neurotoxicity. Ann N Y Acad Sci.

[28] Prinz M, Mildner A (2011). Microglia in the CNS: Immigrants from another world. Glia.

[29] Prinz M, Priller J (2017). The role of peripheral immune cells in the CNS in steady state and disease. Nat Neurosci.

[30] Ransohoff RM (2016). A polarizing question: do M1 and M2 microglia exist?. Nat Neurosci.

[31] Stout RD, Suttles J (2004). Functional plasticity of macrophages: reversible adaptation to changing microenvironments. J Leukoc Biol.

[32] Martinez FO, Gordon S (2014). The M1 and M2 paradigm of macrophage activation: Time for reassessment. F1000Prime Rep..

[33] Murray PJ, Allen JE, Biswas SK, Fisher EA, Gilroy DW, Goerdt S (2014). Macrophage activation and polarization: nomenclature and experimental guidelines. Immunity.

[34] Kierdorf K, Prinz M (2013). Factors regulating microglia activation. Front Cell Neurosci.

[35] Ransohoff RM, Perry VH (2009). Microglial physiology: unique stimuli, specialized responses. Annu Rev Immunol.

[36] Stence N, Waite M, Dailey ME (2001). Dynamics of microglial activation: a confocal time-lapse analysis in hippocampal slices. Glia.

[37] Jurga AM, Paleczna M, Kuter KZ (2020). Overview of general and discriminating markers of differential microglia phenotypes. Front Cell Neurosci.

[38] Bollinger JL, Wohleb ES (2019). The formative role of microglia in stress-induced synaptic deficits and associated behavioral consequences. Neurosci Lett.

[39] Wohleb ES, Franklin T, Iwata M, Duman RS (2016). Integrating neuroimmune systems in the neurobiology of depression. Nat Rev Neurosci.

[40] Denes A, Thornton P, Rothwell NJ, Allan SM (2010). Inflammation and brain injury: acute cerebral ischaemia, peripheral and central inflammation. Brain Behav Immun.

[41] DiSabato DJ, Quan N, Godbout JP (2016). Neuroinflammation: the devil is in the details. J Neurochem.

[42] Zrzavy T, Hametner S, Wimmer I, Butovsky O, Weiner HL, Lassmann H (2017). Loss of “homeostatic” microglia and patterns of their activation in active multiple sclerosis. Brain.

[43] Guerrero BL, Sicotte NL (2020). Microglia in multiple sclerosis: friend or foe?. Front Immunol.

[44] Wheeler DL, Sariol A, Meyerholz DK, Perlman S (2018). Microglia are required for protection against lethal coronavirus encephalitis in mice. J Clin Invest.

[45] Savarin C, Dutta R, Bergmann CC (2018). Distinct gene profiles of bone marrow-derived macrophages and microglia during neurotropic coronavirus-induced demyelination. Front Immunol.

[46] Hatton CF, Duncan CJA (2019). Microglia Are Essential to Protective Antiviral Immunity: Lessons From Mouse Models of Viral Encephalitis. Front Immunol.

[47] Mangale V, Syage AR, Ekiz HA, Skinner DD, Cheng Y, Stone CL (2020). Microglia influence host defense, disease, and repair following murine coronavirus infection of the central nervous system. Glia.

[48] Sarma JD, Burrows A, Rayman P, Hwang MH, Kundu S, Sharma N (2020). Ifit2 deficiency restricts microglial activation and leukocyte migration following murine coronavirus (m-CoV) CNS infection. PLoS Pathog.

[49] Sariol A, Mackin S, Allred MG, Ma C, Zhou Y, Zhang Q (2020). Microglia depletion exacerbates demyelination and impairs remyelination in a neurotropic coronavirus infection. Proc Natl Acad Sci U S A.

[50] Wlodarczyk A, Benmamar-Badel A, Cédile O, Jensen KN, Kramer I, Elsborg NB (2019). CSF1R stimulation promotes increased neuroprotection by CD11c+ microglia in EAE. Front Cell Neurosci.

[51] Nakajima K, Kohsaka S (2004). Microglia: Neuroprotective and neurotrophic cells in the central nervous system. Curr Drug Targets Cardiovasc Haematol Disord.

[52] Chen AQ, Fang Z, Chen XL, Yang S, Zhou YF, Mao L (2019). Microglia-derived TNF-α mediates endothelial necroptosis aggravating blood brain–barrier disruption after ischemic stroke. Cell Death Dis.

[53] Ritzel RM, Patel AR, Grenier JM, Crapser J, Verma R, Jellison ER (2015). Functional differences between microglia and monocytes after ischemic stroke. J Neuroinflammation.

[54] Zhang Y, Li H, Li X, Wu J, Xue T, Wu J (2020). TMEM16F aggravates neuronal loss by mediating microglial phagocytosis of neurons in a rat experimental cerebral ischemia and reperfusion model. Front Immunol.

[55] Yang J, Cao LL, Wang XP, Guo W, Guo RB, Sun YQ (2021). Neuronal extracellular vesicle derived miR-98 prevents salvageable neurons from microglial phagocytosis in acute ischemic stroke. Cell Death Dis.

[56] Neher JJ, Emmrich JV, Fricker M, Mander PK, Théry C, Brown GC (2013). Phagocytosis executes delayed neuronal death after focal brain ischemia. Proc Natl Acad Sci U S A.

[57] Kawabori M, Kacimi R, Kauppinen T, Calosing C, Kim JY, Hsieh CL (2015). Triggering receptor expressed on myeloid cells 2 (TREM2) deficiency attenuates phagocytic activities of microglia and exacerbates ischemic damage in experimental stroke. J Neurosci.

[58] Haynes SE, Hollopeter G, Yang G, Kurpius D, Dailey ME, Gan WB (2006). The P2Y12 receptor regulates microglial activation by extracellular nucleotides. Nat Neurosci.

[59] Swiatkowski P, Murugan M, Eyo UB, Wang Y, Rangaraju S, Oh SB (2016). Activation of microglial P2Y12 receptor is required for outward potassium currents in response to neuronal injury. Neuroscience.

[60] Patel AR, Ritzel R, McCullough LD, Liu F (2013). Microglia and ischemic stroke: A double-edged sword. Int J Physiol Pathophysiol Pharmacol.

[61] Bradshaw EM, Chibnik LB, Keenan BT, Ottoboni L, Raj T, Tang A (2013). CD33 Alzheimer’s disease locus: Altered monocyte function and amyloid biology. Nat Neurosci.

[62] Guerreiro R, Wojtas A, Bras J, Carrasquillo M, Rogaeva E, Majounie E (2013). TREM2 Variants in Alzheimer’s Disease. N Engl J Med.

[63] Mathys H, Davila-Velderrain J, Peng Z, Gao F, Mohammadi S, Young JZ (2019). Single-cell transcriptomic analysis of Alzheimer’s disease. Nature.

[64] Olah M, Menon V, Habib N, Taga MF, Ma Y, Yung CJ (2020). Single cell RNA sequencing of human microglia uncovers a subset associated with Alzheimer’s disease. Nat Commun.

[65] Da Mesquita S, Papadopoulos Z, Dykstra T, Brase L, Farias FG, Wall M (2021). Meningeal lymphatics affect microglia responses and anti-Aβ immunotherapy. Nature.

[66] Zenaro E, Pietronigro E, Bianca VD, Piacentino G, Marongiu L, Budui S (2015). Neutrophils promote Alzheimer’s disease-like pathology and cognitive decline via LFA-1 integrin. Nat Med.

[67] Zilka N, Stozicka Z, Kovac A, Pilipcinec E, Bugos O, Novak M (2009). Human misfolded truncated tau protein promotes activation of microglia and leukocyte infiltration in the transgenic rat model of tauopathy. J Neuroimmunol.

[68] Togo T, Akiyama H, Iseki E, Kondo H, Ikeda K, Kato M (2002). Occurrence of T cells in the brain of Alzheimer’s disease and other neurological diseases. J Neuroimmunol.

[69] Hansen DV, Hanson JE, Sheng M (2018). Microglia in Alzheimer ’ s disease. J Cell Biol.

[70] Wang Y, Cella M, Mallinson K, Ulrich JD, Young KL, Robinette ML (2015). TREM2 lipid sensing sustains the microglial response in an Alzheimer’s disease model. Cell.

[71] Yuan P, Condello C, Keene CD, Wang Y, Bird TD, Paul SM (2016). TREM2 haplodeficiency in mice and humans impairs the microglia barrier function leading to decreased amyloid compaction and severe axonal dystrophy. Neuron.

[72] Wang S, Mustafa M, Yuede CM, Salazar SV, Kong P, Long H (2020). Anti-human TREM2 induces microglia proliferation and reduces pathology in an Alzheimer’s disease model. J Exp Med.

[73] Keren-Shaul H, Spinrad A, Weiner A, Matcovitch-Natan O, Dvir-Szternfeld R, Ulland TK (2017). A unique microglia type associated with restricting development of alzheimer’s disease. Cell.

[74] Rangaraju S, Dammer EB, Raza SA, Rathakrishnan P, Xiao H, Gao T (2018). Identification and therapeutic modulation of a pro-inflammatory subset of disease-associated-microglia in Alzheimer’s disease. Mol Neurodegener.

[75] Hopp S, Lin Y, Oakley D, Roe A, DeVos S, Hanlon D (2018). The role of microglia in processing and spreading of bioactive tau seeds in Alzheimer’s disease. J Neuroinflammation.

[76] Venegas C, Kumar S, Franklin BS, Dierkes T, Brinkschulte R, Tejera D (2017). Microglia-derived ASC specks cross-seed amyloid-β in Alzheimer’s disease. Nature.

[77] Rock RB, Gekker G, Hu S, Sheng WS, Cheeran M, Lokensgard JR (2004). Role of microglia in central nervous system infections. Clin Microbiol Rev.

[78] Scheld WM, Koedel U, Nathan B, Pfister HW (2002). Pathophysiology of bacterial meningitis: Mechanism(s) of neuronal injury. J Infect Dis.

[79] Hanisch UK, Prinz M, Angstwurm K, Husler KG, Kann O, Kettenmann H (2001). The protein tyrosine kinase inhibitor AG126 prevents the massive microglial cytokine induction by pneumococcal cell walls. Eur J Immunol.

[80] Häusler KG, Prinz M, Nolte C, Weber JR, Schumann RR, Kettenmann H (2002). Interferon-γ differentially modulates the release of cytokines and chemokines in lipopolysaccharide- and pneumococcal cell wall-stimulated mouse microglia and macrophages. Eur J Neurosci.

[81] Ring A, Weiser JN, Tuomanen EI (1998). Pneumococcal trafficking across the blood-brain barrier molecular analysis of a novel bidirectional pathway. J Clin Invest.

[82] Kim YS, Täuber MG (1996). Neurotoxicity of glia activated by gram-positive bacterial products depends on nitric oxide production. Infect Immun.

[83] Skiest DJ (2002). Focal neurological disease in patients with acquired immunodeficiency syndrome. Clin Infect Dis.

[84] Braun JS, Sublett JE, Freyer D, Mitchell TJ, Cleveland JL, Tuomanen EI (2002). Pneumococcal pneumolysin and H2O2 mediate brain cell apoptosis during meningitis. J Clin Invest.

[85] Schiltz JC, Sawchenko PE (2002). Distinct brain vascular cell types manifest inducible cyclooxygenase expression as a function of the strength and nature of immune insults. J Neurosci.

[86] Quan N, Whiteside M, Herkenham M (1998). Time course and localization patterns of interleukin-1β messenger RNA expression in brain and pituitary after peripheral administration of lipopolysaccharide. Neuroscience.

[87] Konsman JP, Vigues S, Mackerlova L, Bristow A, Blomqvist A (2004). Rat Brain Vascular Distribution of Interleukin-1 Type-1 Receptor Immunoreactivity: Relationship to Patterns of Inducible Cyclooxygenase Expression by Peripheral Inflammatory Stimuli. J Comp Neurol.

[88] Dantzer R, O’Connor JC, Freund GG, Johnson RW, Kelley KW (2008). From inflammation to sickness and depression: When the immune system subjugates the brain. Nat Rev Neurosci.

[89] Norden DM, Trojanowski PJ, Villanueva E, Navarro E, Godbout JP (2016). Sequential activation of microglia and astrocyte cytokine expression precedes increased iba-1 or GFAP immunoreactivity following systemic immune challenge. Glia.

[90] Zeng J, Zhào H, Liu Z, Zhang W, Huang Y (2018). Lipopolysaccharide Induces Subacute Cerebral Microhemorrhages with Involvement of Nitric Oxide Synthase in Rats. J Stroke Cerebrovasc Dis.

[91] Sardari M, Skuljec J, Yin D, Zec K, de Carvalho TS, Albers D (2021). Lipopolysaccharide-induced sepsis-like state compromises post-ischemic neurological recovery, brain tissue survival and remodeling via mechanisms involving microvascular thrombosis and brain T cell infiltration. Brain Behav Immun.

[92] Sumbria RK, Grigoryan MM, Vasilevko V, Krasieva TB, Scadeng M, Dvornikova AK (2016). A murine model of inflammation-induced cerebral microbleeds. J Neuroinflammation.

[93] Banks WA, Robinson SM (2010). Minimal penetration of lipopolysaccharide across the murine blood-brain barrier. Brain Behav Immun.

[94] Nimmerjahn A, Kirchhoff F, Helmchen F (2005). Resting microglial cells are highly dynamic surveillants of brain parenchyma in vivo. Science.

[95] Davalos D, Grutzendler J, Yang G, Kim JV, Zuo Y, Jung S (2005). ATP mediates rapid microglial response to local brain injury in vivo. Nat Neurosci.

[96] Katz LC, Shatz CJ (1996). Synaptic activity and the construction of cortical circuits. Science.

[97] Hua JY, Smith SJ (2004). Neural activity and the dynamics of central nervous system development. Nat Neurosci.

[98] Stevens B, Allen NJ, Vazquez LE, Howell GR, Christopherson KS, Nouri N (2007). The classical complement cascade mediates CNS synapse elimination. Cell.

[99] Medzhitov R, Janeway CA (2002). Decoding the patterns of self and nonself by the innate immune system. Science.

[100] Carroll MC (2004). The complement system in regulation of adaptive immunity. Nat Immunol.

[101] Zipfel PF, Skerka C (2009). Complement regulators and inhibitory proteins. Nat Rev Immunol.

[102] Ricklin D, Hajishengallis G, Yang K, Lambris JD (2010). Complement: A key system for immune surveillance and homeostasis. Nat Immunol.

[103] Schafer DP, Lehrman EK, Kautzman AG, Koyama R, Mardinly AR, Yamasaki R (2012). Microglia sculpt postnatal neural circuits in an activity and complement-dependent manner. Neuron.

[104] Weinhard L, Di Bartolomei G, Bolasco G, Machado P, Schieber NL, Neniskyte U (2018). Microglia remodel synapses by presynaptic trogocytosis and spine head filopodia induction. Nat Commun.

[105] Sipe GO, Lowery RL, Tremblay M, Kelly EA, Lamantia CE, Majewska AK (2016). Microglial P2Y12 is necessary for synaptic plasticity in mouse visual cortex. Nat Commun.

[106] Gunner G, Cheadle L, Johnson KM, Ayata P, Badimon A, Mondo E (2019). Sensory lesioning induces microglial synapse elimination via ADAM10 and fractalkine signaling. Nat Neurosci.

[107] Filipello F, Morini R, Corradini I, Zerbi V, Canzi A, Michalski B (2018). The Microglial innate immune receptor TREM2 is required for synapse elimination and normal brain connectivity. Immunity.

[108] Paolicelli RC, Bolasco G, Pagani F, Maggi L, Scianni M, Panzanelli P (2011). Synaptic pruning by microglia is necessary for normal brain development. Science.

[109] Rogers JT, Morganti JM, Bachstetter AD, Hudson CE, Peters MM, Grimmig BA (2011). CX3CR1 deficiency leads to impairment of hippocampal cognitive function and synaptic plasticity. J Neurosci.

[110] Wang C, Yue H, Hu Z, Shen Y, Ma J, Li J (2020). Microglia mediate forgetting via complement-dependent synaptic elimination. Science.

[111] Eyo UB, Wu L-J (2013). Bidirectional microglia-neuron communication in the healthy brain. Neural Plast.

[112] Dissing-Olesen L, LeDue JM, Rungta RL, Hefendehl JK, Choi HB, MacVicar BA (2014). Activation of neuronal NMDA receptors triggers transient ATP-mediated microglial process outgrowth. J Neurosci.

[113] Eyo UB, Gu N, De S, Dong H, Richardson JR, Wu L-J (2015). Modulation of microglial process convergence toward neuronal dendrites by extracellular calcium. J Neurosci.

[114] Pfeiffer T, Avignone E, Nägerl UV (2016). Induction of hippocampal long-term potentiation increases the morphological dynamics of microglial processes and prolongs their contacts with dendritic spines. Sci Rep.

[115] Eyo UB, Bispo A, Liu J, Sabu S, Wu R, Dibona VL (2018). The GluN2A subunit regulates neuronal NMDA receptor-induced microglia-neuron physical interactions. Sci Rep.

[116] Bernier L-P, Bohlen CJ, York EM, Stevens B, Barres BA, Correspondence BAM (2019). Nanoscale surveillance of the brain by microglia via cAMP-regulated filopodia. Cell Rep.

[117] Tremblay MĚ, Lowery RL, Majewska AK (2010). Microglial interactions with synapses are modulated by visual experience. PLoS Biol.

[118] Stowell RD, Sipe GO, Dawes RP, Batchelor HN, Lordy KA, Whitelaw BS (2019). Noradrenergic signaling in the wakeful state inhibits microglial surveillance and synaptic plasticity in the mouse visual cortex. Nat Neurosci.

[119] Badimon A, Strasburger HJ, Ayata P, Chen X, Nair A, Ikegami A (2020). Negative feedback control of neuronal activity by microglia. Nature.

[120] Schneider H, Pitossi F, Balschun D, Wagner A, Del Rey A, Besedovsky HO (1998). A neuromodulatory role of interleukin-1β in the hippocampus. Proc Natl Acad Sci U S A.

[121] Spulber S, Mateos L, Oprica M, Cedazo-Minguez A, Bartfai T, Winblad B (2009). Impaired long term memory consolidation in transgenic mice overexpressing the human soluble form of IL-1ra in the brain. J Neuroimmunol.

[122] Prieto GA, Cotman CW (2017). Cytokines and cytokine networks target neurons to modulate long-term potentiation. Cytokine Growth Factor Rev.

[123] Ross FM, Allan SM, Rothwell NJ, Verkhratsky A (2003). A dual role for interleukin-1 in LTP in mouse hippocampal slices. J Neuroimmunol.

[124] Goshen I, Kreisel T, Ounallah-Saad H, Renbaum P, Zalzstein Y, Ben-Hur T (2007). A dual role for interleukin-1 in hippocampal-dependent memory processes. Psychoneuroendocrinology.

[125] Prieto GA, Snigdha S, Baglietto-Vargas D, Smith ED, Berchtold NC, Tong L (2015). Synapse-specific IL-1 receptor subunit reconfiguration augments vulnerability to IL-1β in the aged hippocampus. Proc Natl Acad Sci.

[126] Di Castro MA, Trettel F, Milior G, Maggi L, Ragozzino D, Limatola C (2016). The chemokine CXCL16 modulates neurotransmitter release in hippocampal CA1 area. Sci Rep.

[127] Ragozzino D, Di Angelantonio S, Trettel F, Bertollini C, Maggi L, Gross C (2006). Chemokine fractalkine/CX3CL1 negatively modulates active glutamatergic synapses in rat hippocampal neurons. J Neurosci.

[128] Lim SH, Park E, You B, Jung Y, Park AR, Park SG (2013). Neuronal synapse formation induced by microglia and interleukin 10. PLoS ONE.

[129] Maggi L, Trettel F, Scianni M, Bertollini C, Eusebi F, Fredholm BB (2009). LTP impairment by fractalkine/CX3CL1 in mouse hippocampus is mediated through the activity of adenosine receptor type 3 (A3R). J Neuroimmunol.

[130] Bertollini C, Ragozzino D, Gross C, Limatola C, Eusebi F (2006). Fractalkine/CX3CL1 depresses central synaptic transmission in mouse hippocampal slices. Neuropharmacology.

[131] Stellwagen D, Malenka RC (2006). Synaptic scaling mediated by glial TNF-α. Nature.

[132] Wohleb ES (2016). Neuron-microglia interactions in mental health disorders: “For better, and for worse”. Front Immunol.

[133] Nguyen KT, Deak T, Owens SM, Kohno T, Fleshner M, Watkins LR (1998). Exposure to acute stress induces brain interleukin-1β protein in the rat. J Neurosci.

[134] Goshen I, Kreisel T, Ben-Menachem-Zidon O, Licht T, Weidenfeld J, Ben-Hur T (2008). Brain interleukin-1 mediates chronic stress-induced depression in mice via adrenocortical activation and hippocampal neurogenesis suppression. Mol Psychiatry.

[135] Nguyen KT, Deak T, Will MJ, Hansen MK, Hunsaker BN, Fleshner M (2000). Timecourse and corticosterone sensitivity of the brain, pituitary, and serum interleukin-1β protein response to acute stress. Brain Res.

[136] Tynan RJ, Naicker S, Hinwood M, Nalivaiko E, Buller KM, Pow DV (2010). Chronic stress alters the density and morphology of microglia in a subset of stress-responsive brain regions. Brain Behav Immun.

[137] Blandino P, Barnum CJ, Deak T (2006). The involvement of norepinephrine and microglia in hypothalamic and splenic IL-1β responses to stress. J Neuroimmunol.

[138] Johnson JD, Campisi J, Sharkey CM, Kennedy SL, Nickerson M, Greenwood BN (2005). Catecholamines mediate stress-induced increases in peripheral and central inflammatory cytokines. Neuroscience.

[139] Saito K, Suyama K, Nishida K, Sei Y, Basile AS (1996). Early increases in TNFa, IL-6 and IL-1b levels following transient cerebral ischemia in gerbil brain. Neurosci Lett.

[140] Barichello T, dos Santos I, Savi GD, Florentino AF, Silvestre C, Comim CM (2009). Tumor necrosis factor alpha (TNF-α) levels in the brain and cerebrospinal fluid after meningitis induced by Streptococcus pneumoniae. Neurosci Lett.

[141] Barichello T, dos Santos I, Savi GD, Simões LR, Silvestre T, Comim CM (2010). TNF-α, IL-1β, IL-6, and cinc-1 levels in rat brain after meningitis induced by Streptococcus pneumoniae. J Neuroimmunol.

[142] Grundy RI, Rothwell NJ, Allan SM (1999). Dissociation between the effects of interleukin-1 on excitotoxic brain damage and body temperature in the rat. Brain Res.

[143] Sedger LM, McDermott MF (2014). TNF and TNF-receptors: from mediators of cell death and inflammation to therapeutic giants—past, present and future. Cytokine Growth Factor Rev.

[144] Wooff Y, Man SM, Aggio-Bruce R, Natoli R, Fernando N (2019). IL-1 family members mediate cell death, inflammation and angiogenesis in retinal degenerative diseases. Front Immunol.

[145] O’Connor KA, Johnson JD, Hansen MK, Wieseler Frank JL, Maksimova E, Watkins LR (2003). Peripheral and central proinflammatory cytokine response to a severe acute stressor. Brain Res.

[146] Walker AK, Nakamura T, Hodgson DM (2010). Neonatal lipopolysaccharide exposure alters central cytokine responses to stress in adulthood in Wistar rats. Stress.

[147] Yirmiya R, Goshen I (2011). Immune modulation of learning, memory, neural plasticity and neurogenesis. Brain Behav Immun.

[148] Lewitus GM, Pribiag H, Duseja R, St-Hilaire M, Stellwagen D (2014). An adaptive role of TNF in the regulation of striatal synapses. J Neurosci.

[149] Li Q, Barres BA (2018). Microglia and macrophages in brain homeostasis and disease. Nat Rev Immunol.

[150] Ueno M, Fujita Y, Tanaka T, Nakamura Y, Kikuta J, Ishii M (2013). Layer v cortical neurons require microglial support for survival during postnatal development. Nat Neurosci.

[151] Shigemoto-Mogami Y, Hoshikawa K, Goldman JE, Sekino Y, Sato K (2014). Microglia enhance neurogenesis and oligodendrogenesis in the early postnatal subventricular zone. J Neurosci.

[152] Woodburn SC, Bollinger JL, Wohleb ES (2021). Synaptic and behavioral effects of chronic stress are linked to dynamic and sex-specific changes in microglia function and astrocyte dystrophy. Neurobiol Stress.

[153] Bollinger JL, Burns CMB, Wellman CL (2016). Differential effects of stress on microglial cell activation in male and female medial prefrontal cortex. Brain Behav Immun.

[154] Horchar MJ, Wohleb ES (2019). Glucocorticoid receptor antagonism prevents microglia-mediated neuronal remodeling and behavioral despair following chronic unpredictable stress. Brain Behav Immun.

[155] Bollinger JL, Horchar MJ, Wohleb ES (2020). Diazepam limits microglia-mediated neuronal remodeling in the prefrontal cortex and associated behavioral consequences following chronic unpredictable stress. Neuropsychopharmacology.

[156] Kopp BL, Wick D, Herman JP (2013). Differential effects of homotypic vs heterotypic chronic stress regimens on microglial activation in the prefrontal cortex. Physiol Behav.

[157] Lehmann ML, Cooper HA, Maric D, Herkenham M (2016). Social defeat induces depressive-like states and microglial activation without involvement of peripheral macrophages. J Neuroinflammation.

[158] Mackaness GB (1962). Cellular Resistance to Infection. J Exp Med.

[159] Wohleb ES, Powell ND, Godbout JP, Sheridan JF (2013). Stress-induced recruitment of bone marrow-derived monocytes to the brain promotes anxiety-like behavior. J Neurosci.

[160] Wohleb ES, Patterson JM, Sharma V, Quan N, Godbout JP, Sheridan JF (2014). Knockdown of interleukin-1 receptor type-1 on endothelial cells attenuated stress-induced neuroinflammation and prevented anxiety-like behavior. J Neurosci.

[161] Wohleb ES, McKim DB, Shea DT, Powell ND, Tarr AJ, Sheridan JF (2014). Re-establishment of anxiety in stress-sensitized mice is caused by monocyte trafficking from the spleen to the brain. Biol Psychiatry.

[162] Lehmann ML, Weigel TK, Cooper HA, Elkahloun AG, Kigar SL, Herkenham M (2018). Decoding microglia responses to psychosocial stress reveals blood-brain barrier breakdown that may drive stress susceptibility. Sci Rep.

[163] Menard C, Pfau ML, Hodes GE, Kana V, Wang VX, Bouchard S (2017). Social stress induces neurovascular pathology promoting depression. Nat Neurosci.

[164] Wilkinson FL, Sergijenko A, Langford-Smith KJ, Malinowska M, Wynn RF, Bigger BW (2013). Busulfan conditioning enhances engraftment of hematopoietic donor-derived cells in the brain compared with irradiation. Mol Ther.

[165] Lehmann ML, Poffenberger CN, Elkahloun AG, Herkenham M (2020). Analysis of cerebrovascular dysfunction caused by chronic social defeat in mice. Brain Behav Immun.

[166] Sial OK, Warren BL, Alcantara LF, Parise EM, Bolaños-Guzmán CA (2016). Vicarious social defeat stress: Bridging the gap between physical and emotional stress. J Neurosci Methods.

[167] Golden SA, Covington HE, Berton O, Russo SJ (2011). A standardized protocol for repeated social defeat stress in mice. Nat Protoc.

[168] Xu Z, Dong Y, Wang H, Culley DJ, Marcantonio ER, Crosby G (2014). Peripheral surgical wounding and age-dependent neuroinflammation in mice. PLoS ONE.

[169] Wohleb ES, Terwilliger R, Duman CH, Duman RS (2018). Stress-Induced Neuronal Colony Stimulating Factor 1 Provokes Microglia-Mediated Neuronal Remodeling and Depressive-like Behavior. Biol Psychiatry.

[170] Herman JP (2013). Neural control of chronic stress adaptation. Front Behav Neurosci.

[171] Ulrich-Lai YM, Herman JP (2009). Neural regulation of endocrine and autonomic stress responses. Nat Rev Neurosci.

[172] Luczynski P, Moquin L, Gratton A (2015). Chronic stress alters the dendritic morphology of callosal neurons and the acute glutamate stress response in the rat medial prefrontal cortex. Stress.

[173] Ayash S, Schmitt U, Müller MB (2020). Chronic social defeat-induced social avoidance as a proxy of stress resilience in mice involves conditioned learning. J Psychiatr Res.

[174] Hawkins J, Hicks RA, Phillips N, Moore JD (1978). Swimming rats and human depression. Nature.

[175] Riga D, Theijs JT, De Vries TJ, Smit AB, Spijker S (2015). Social defeat-induced anhedonia: effects on operant sucrose-seeking behavior. Front Behav Neurosci.

[176] Dieterich A, Srivastava P, Sharif A, Stech K, Floeder J, Yohn SE (2019). Chronic corticosterone administration induces negative valence and impairs positive valence behaviors in mice. Transl Psychiatry.

[177] Dieterich A, Stech K, Srivastava P, Lee J, Sharif A, Samuels BA (2020). Chronic corticosterone shifts effort-related choice behavior in male mice. Psychopharmacology.

[178] Alves-dos-Santos L, de Resende LS, Chiavegatto S (2020). Susceptibility and resilience to chronic social defeat stress in adolescent male mice: No correlation between social avoidance and sucrose preference. Neurobiol Stress.

[179] Yuen EY, Wei J, Liu W, Zhong P, Li X, Yan Z (2012). Repeated stress causes cognitive impairment by suppressing glutamate receptor expression and function in prefrontal cortex. Neuron.

[180] Tauber AI (2003). Metchnikoff and the phagocytosis theory. Nat Rev Mol Cell Biol.

[181] Medzhitov R (2008). Origin and physiological roles of inflammation. Nature.

[182] Liu T, Lu J, Lukasiewicz K, Pan B, Zuo Y (2021). Stress induces microglia-associated synaptic circuit alterations in the dorsomedial prefrontal cortex. Neurobiol Stress..

[183] Nair A, Bonneau RH (2006). Stress-induced elevation of glucocorticoids increases microglia proliferation through NMDA receptor activation. J Neuroimmunol.

[184] Sugama S, Takenouchi T, Fujita M, Conti B, Hashimoto M (2009). Differential microglial activation between acute stress and lipopolysaccharide treatment. J Neuroimmunol.

[185] Zhou L-J, Peng J, Xu Y-N, Tan Z, Liu X-G (2019). Microglia are indispensable for synaptic plasticity in the spinal dorsal horn and chronic pain. Cell Rep.

[186] Luo J, Elwood F, Britschgi M, Villeda S, Zhang H, Ding Z (2013). Colony-stimulating factor 1 receptor (CSF1R) signaling in injured neurons facilitates protection and survival. J Exp Med.

[187] Yuen EY, Liu W, Karatsoreos IN, Feng J, McEwen BS, Yan Z (2009). Acute stress enhances glutamatergic transmission in prefrontal cortex and facilitates working memory. Proc Natl Acad Sci U S A.

[188] Bagley J, Moghaddam B (1997). Temporal dynamics of glutamate efflux in the prefrontal cortex and in the hippocampus following repeated stress: effects of pretreatment with saline or diazepam:. Neuroscience.

[189] Moghaddam B, Bolinao ML, Stein-Behrens B, Sapolsky R (1994). Glucocortcoids mediate the stress-induced extracellular accumulation of glutamate. Brain Res.

[190] Popoli M, Yan Z, McEwen BS, Sanacora G (2012). The stressed synapse: The impact of stress and glucocorticoids on glutamate transmission. Nat Rev Neurosci.

[191] McEwen BS, Bowles NP, Gray JD, Hill MN, Hunter RG, Karatsoreos IN (2015). Mechanisms of stress in the brain. Nat Neurosci.

[192] McEwen BS (2000). Allostasis, allostatic load, and the aging nervous system: role of excitatory amino acids and excitotoxicity. Neurochem Res.

[193] Sapolsky RM (1994). Glucocorticoids, stress and exacerbation of excitotoxic neuron death. Semin Neurosci.

[194] Zoppi S, Pérez Nievas BG, Madrigal JLM, Manzanares J, Leza JC, García-Bueno B (2011). Regulatory role of cannabinoid receptor 1 in stress-induced excitotoxicity and neuroinflammation. Neuropsychopharmacology.

[195] Takahashi T, Kimoto T, Tanabe N, Hattori T, Yasumatsu N, Kawato S (2002). Corticosterone acutely prolonged N-methyl-D-aspartate receptor-mediated Ca2+ elevation in cultured rat hippocampal neurons. J Neurochem.

[196] Liu R, Aghajanian GK (2008). Stress blunts serotonin- and hypocretin-evoked EPSCs in prefrontal cortex: Role of corticosterone-mediated apical dendritic atrophy. Proc Natl Acad Sci.

[197] Cook SC, Wellman CL (2004). Chronic stress alters dendritic morphology in rat medial prefrontal cortex. J Neurobiol.

[198] Wellman CL (2001). Dendritic reorganization in pyramidal neurons in medial prefrontal cortex after chronic corticosterone administration. Corticosterone Prefrontal Morphol.

[199] Holmes A, Wellman CL (2009). Stress-induced prefrontal reorganization and executive dysfunction in rodents. Neurosci Biobehav Rev.

[200] Radley JJ, Rocher AB, Miller M, Janssen WGM, Liston C, Hof PR (2006). Repeated stress induces dendritic spine loss in the rat medial prefrontal cortex. Cereb Cortex.

[201] Goldwater DS, Pavlides C, Hunter RG, Bloss EB, Hof PR, McEwen BS (2009). Structural and functional alterations to rat medial prefrontal cortex following chronic restraint stress and recovery. Neuroscience.

[202] Frank MG, Baratta MV, Sprunger DB, Watkins LR, Maier SF (2007). Microglia serve as a neuroimmune substrate for stress-induced potentiation of CNS pro-inflammatory cytokine responses. Brain Behav Immun.

[203] Moda-Sava RN, Murdock MH, Parekh PK, Fetcho RN, Huang BS, Huynh TN (2019). Sustained rescue of prefrontal circuit dysfunction by antidepressant-induced spine formation. Science.

[204] Setiawan E, Wilson AA, Mizrahi R, Rusjan PM, Miler L, Rajkowska G (2015). Role of translocator protein density, a marker of neuroinflammation, in the brain during major depressive episodes. JAMA Psychiat.

[205] Dowlati Y, Herrmann N, Swardfager W, Liu H, Sham L, Reim EK (2010). A meta-analysis of cytokines in major depression. Biol Psychiatry.

[206] Prasad AS, Fitzgerald JT, Bao B, Beck FWJ, Chandrasekar PH (2000). Duration of symptoms and plasma cytokine levels in patients with the common cold treated with zinc acetate: A randomized, double-blind, placebo-controlled trial. Ann Intern Med.

[207] Kemna E, Pickkers P, Nemeth E, Van Der Hoeven H, Swinkels D (2005). Time-course analysis of hepcidin, serum iron, and plasma cytokine levels in humans injected with LPS. Blood.

[208] Koch RM, Kox M, Thijs EJM, Rahamat-Langendoen JC, van de Veerdonk FL, Gerretsen J (2017). Development of endotoxin tolerance does not influence the response to a challenge with the mucosal live-attenuated influenza vaccine in humans in vivo. Front Immunol.

[209] Werry EL, Bright FM, Piguet O, Ittner LM, Halliday GM, Hodges JR (2019). Recent developments in TSPO PET imaging as a biomarker of neuroinflammation in neurodegenerative disorders. Int J Mol Sci.

[210] Nutma E, Gebro E, Marzin MC, Valk P, Matthews PM, Owen DR (2021). Activated microglia do not increase 18 kDa translocator protein ( TSPO ) expression in the multiple sclerosis brain. Glia.

[211] Rizzo G, Veronese M, Tonietto M, Bodini B, Stankoff B, Wimberley C (2019). Generalization of endothelial modelling of TSPO PET imaging: considerations on tracer affinities. J Cereb Blood Flow Metab.

[212] Coughlin JM, Yuchuanwang Y, Minn I, Bienko N, Ambinder EB, Xu X (2017). Imaging of glial cell activation and white matter integrity in brains of active and recently retired national football league players. JAMA Neurol.

[213] Notter T, Schalbetter SM, Clifton NE, Mattei D, Richetto J, Thomas K (2020). Neuronal activity increases translocator protein (TSPO) levels. Mol Psychiatry.

[214] Bottcher C, Fernandez-Zapata C, Snijders GJ, Schlickeiser S, Sneeboer MAM, Kunkel D (2020). Single-cell mass cytometry of microglia in major depressive disorder reveals a non-inflammatory phenotype with increased homeostatic marker expression. Transl Psychiatry.

[215] Capuron L, Hauser P, Hinze-selch D, Miller AH, Neveu PJ (2002). Treatment of cytokine-induced depression. Brain Behav Immun.

[216] Breitbart W, Rosenfeld B, Tobias K, Pessin H, Ku GY, Yuan J (2014). Depression, cytokines, and pancreatic cancer. Psychooncology.

[217] Turvey CL, Schultz SK, Beglinger L, Klein DM (2009). A longitudinal community-based study of chronic illness, cognitive and physical function, and depression. Am J Geriatr Psychiatry.

[218] Lynall ME, Turner L, Bhatti J, Cavanagh J, de Boer P, Mondelli V (2020). Peripheral blood cell-stratified subgroups of inflamed depression. Biol Psychiatry.

[219] Price RB, Duman R (2019). Neuroplasticity in cognitive and psychological mechanisms of depression: an integrative model. Mol Psychiatry.

[220] Borsboom D, Cramer AOJ, Kalis A (2019). Brain disorders? Not really: why network structures block reductionism in psychopathology research. Behav Brain Sci.

[221] Beck AT, Bredemeier K (2016). A unified model of depression: Integrating clinical, cognitive, biological, and evolutionary perspectives. Clin Psychol Sci.

[222] Bienertova-Vasku J, Lenart P, Scheringer M (2020). Eustress and distress: neither good nor bad, but rather the same?. BioEssays.

[223] Brachman RA, Lehmann ML, Maric D, Herkenham M (2015). Lymphocytes from chronically stressed mice confer antidepressant-like effects to naive mice. J Neurosci.

[224] Sankowski R, Böttcher C, Masuda T, Geirsdottir L, Sagar SE (2019). Mapping microglia states in the human brain through the integration of high-dimensional techniques. Nat Neurosci.

[225] Masuda T, Sankowski R, Staszewski O, Böttcher C, Sagar AL (2019). Spatial and temporal heterogeneity of mouse and human microglia at single-cell resolution. Nature.

